# Gene polymorphism and protein of human pro- and anti-inflammatory cytokines in Chinese healthy subjects and chronic periodontitis patients

**DOI:** 10.1186/1479-5876-10-S1-S8

**Published:** 2012-09-19

**Authors:** Wings TY Loo, Chang-bin Fan, Lan-jun Bai, Yuan Yue, Yi-ding Dou, Min Wang, Hao Liang, Mary NB Cheung, Louis WC Chow, Jin-le Li, Ye Tian, Liu Qing

**Affiliations:** 1 UNIMED Medical Institute, Hong Kong SAR; 2 School of Chinese Medicine, Li Ka Shing Faculty of Medicine, The University of Hong Kong, Hong Kong SAR; 3 Stomatological Hospital of Guangzhou Medical College, Guangzhou, PRC; 4 Department of Stomatology, Sichuan Academy of Medical Sciences & Sichuan Provincial People’s Hospital, No.32, Section 2,1st Ring Road (West), Chengdu, Sichuan, PRC; 5 State Key Laboratory for Oral Diseases and Department of Prosthodontics, West China Hospital of Stomatology, Sichuan University, Sichuan, PRC; 6 Jin Hua Dentistry, Chengdu, 610041, Sichuan, PRC

## Abstract

**Background:**

Periodontal disease is thought to arise from the interaction of various factors, including the susceptibility of the host, the presence of pathogenic organisms, and the absence of beneficial species. The genetic factors may play a significant role in the risk of periodontal diseases. Cytokines initiate, mediate and control immune and inflammatory responses. The aim of this study is to compare genotypes and soluble protein of pro and anti-inflammatory cytokines (IL-1α, IL-1β, IL-6, IFN-γ, IL-10, TNF-α and IL-4) in subjects with or free of chronic periodontitis.

**Methods:**

A total of 1,290 Chinese subjects were recruited to this clinical trial: 850 periodontally healthy controls and 440 periodontal patients. All subjects were free of systemic diseases. Oral examinations were performed, and the following parameters were recorded for each subject: supragingival/subgingival calculus, gingival recession, bleeding on probing (BOP), probing depth (PD), clinical attachment loss (CAL), gingival recession and tooth mobility. The peripheral blood samples were collected for genetic and enzyme linked immunosorbent assay (ELISA) analysis. Restriction enzymes were used for digestion of amplified fragments of IL-1α, IL-1β, IL-6, IFN-γ, IL-10, TNF-α and IL-4.

**Results:**

The protein expressions of patient and control samples for IL-1α, IL-1β, IL-6, TNF-α, IFN-γ, IL-10, and IL-4 measured by ELISA confirmed a statistically significant difference (p < 0.001). The digestion of fragments of various genes showed that the pro-inflammatory cytokines IL-1α and TNF-α, and the anti-inflammatory cytokines IL-4 and IL-10 demonstrated a correlation with chronic inflammation in patients (*X^2^*: p < 0.001). The remaining genes investigated in patients and healthy subjects (IL-1β, IL-6, IFN-γ and IL-10) did not show any significant difference.

**Conclusions:**

The cytokine gene polymorphisms may be used as a marker for periodontitis susceptibility, clinical behaviour and severity. This detection offers early diagnosis and induction of prophylaxis to other family members against disease progression.

## Background

Periodontal disease is thought to arise from the interaction of various factors, including the susceptibility of the host, the presence of pathogenic organisms and the absence of beneficial species [1]. Although bacteria cause plaque-induced inflammatory periodontal diseases, progression and clinical characteristics of these diseases are influenced by both acquired and genetic factors that can modify susceptibility to infection [2] Reports have indicated that genetic factors may play a significant role in the risk of periodontal diseases [3,4].

Cytokines are soluble proteins which are secreted by cells to act as a messenger that transmits signals to other cells. They initiate, mediate and control immune and inflammatory responses; they also regulate growth and differentiation of cells [4]. Gingival epithelial cells produce a broad range of cytokines, among which, interleukin-1α (IL-1α), interleukin-1β (IL-1β), tumour necrosis factor-α (TNF-α), interleukin-6 (IL-6) and interferon-γ (IFN-γ) are the pro-inflammatory cytokines whereas interleukin-4 (IL-4) and interleukin-10 (IL-10) are the anti-inflammatory cytokines [5-7].

IL-1 consists of at least two separate gene products, IL-1α and IL-1β, which have common biological activities but limited homology at the nucleotide and peptide levels [8]. The gene encoding IL-1 is assigned to chromosome 2q13–21 [9]. The carriage of certain alleles of IL-1α and IL-1β is associated with the incidence and the severity of periodontal disease, in particular chronic periodontitis (CP), because these carriers produce more IL-1 in response to plaque than genotype negative individuals [2,10,11]. The presence of a single base-pair C-T tranpositional polymorphism upstream of the IL-1α gene at position -889 affects the production of the IL-1α protein. The IL-1 α -889 T allele was associated with an almost four-fold increase in IL-1 α levels in gingival crevicular fluid [12]. A weak positive association was found concerning IL-1β T(-511)C and chronic periodontal disease [13]. In other studies, no association was found for IL-1α C(-889)T and -1β T(-511)C with aggressive periodontitis [13,14].

TNF-α is located in 6p21.3 of chromosome 6 within the major histocompatibility complex [15]. Eight single nucleotide polymorphisms (SNPs) in the promoter region of this gene have been studied at positions -1031T/C, -863C/A, -857C/T, -575G/A, -376G/A, -308G/A, -244G/A, and -238G/A [16-19]. Many researchers investigated the possible link between the -308 polymorphism in the TNF-α gene and periodontitis because a G to A polymorphism at the -308 position of the TNF-α promoter region was suggested to influence TNF-α production and monocytes of patients with periodontitis [20,21]. Higher allele 2 frequency was observed in patients with chronic periodontitis than in healthy individuals [22]. Aggressive periodontitis was not linked to this locus [23-25]. The remaining promoter polymorphisms were not associated with periodontitis, apart from one study that found an association between the -1031, -863, -857 SNPs and chronic periodontitis severity in the Japanese population [23,26,27].

The IL-6 gene is assigned to chromosome 7p21. Various SNPs in the promoter region of this gene have been studied at positions -174G/C, -190C/T, -572C/G, -597G/A, -1363G/T, -1480C/G and -6106A/T [28,29]. In periodontitis, the G–C SNP at the -174 position correlated with chronic periodontitis susceptibility in Brazilians and Caucasians, but not the Japanese [30,31]. Periodontitis patients carrying one or two copies of the rare allele in the IL-6 -174 polymorphism displayed significantly higher serum IL-6 and C-reactive protein concentrations [32]. Carriers of the rare allele at this position was associated with less reduction in probing depths among chronic periodontitis patients after delivery of standard non-surgical periodontal therapy [33]. On the contrary, another study found that the -174 R-allele carrier individuals have decreased plasma levels of IL-6 and lower IL-6 gene transcriptional activity compared with N/N individuals, suggesting that low IL-6 response may hamper an individual’s defense against periodontal pathogens [34]. In a meta-analysis of cytokine gene polymorphisms of 53 studies, the IL-6 -174 polymorphism did not exhibit any association with chronic periodontitis (CP) [13].

The mRNA expression and/or concentration of IFN-γ in gingival crevicular fluid, gingival tissues, and serum were able to affect gingivitis, probing depths and alveolar bone loss [35]. Very few studies have investigated the connection of polymorphism of IFN-γ with periodontitis to date. Polymorphism in gene IFN-γ was found to be functionally relevant and causes differences in the immunoregulatory activity of its cytokine molecules. The T allele of the IFN-γ 874 T/A is found in high producers of IFN-γ [36]. No association was found in the only existing study dealing with the IFN- γ polymorphism 874T/A and CP [37].

The gene for IL-4 is localized in chromosome 5q31.1 [38]. The presence of IL-4-producing cells and the percentage of IL-4-expressing cells were significantly higher in established and severe periodontitis lesions than in gingivitis tissues. IL-4 levels in the serum of patients were higher in chronic periodontitis but these levels did not correlate with the degree of bone loss or pocket formation [39]. Mout *et al.* identified promoter SNP at position -590 and a 70-bp variable numbers of tandem repeat (VNTR) polymorphism at intron 2 [40]. However, many reports have failed to establish a connection between these loci and periodontal disease susceptibility and severity [41,42].

The gene encoding IL-10 was mapped to chromosome 1q31–32 [43]. The -1082 G/A locus was not associated with chronic periodontitis susceptibility in most Caucasian populations except in one Swedish study, but was linked to CP severity [44]. The -1082 single nucleotide polymorphism was associated with high *in vitro* IL-10 production [45]. There was a complete absence of the N-allele carriage at position -1082 among the Japanese in contrast to Caucasians where the -1082 N-allele is the most occurring variant [45,46].

The aim of this study is to compare genotypes and soluble protein (serum) of pro and anti-inflammatory cytokines in subjects with or free of chroinic periodontitis.

## Methods

### Selection of subjects and clinical examination

From September 2004 to March 2007, a total of 1,290 subjects were recruited to this clinical trial: 850 periodontally healthy subjects and 440 periodontitis patients. Healthy subjects were selected from colleges and the community setting to be in the control group. They were invited for oral clinical examinations at West China Hospital of Stomatology, Sichuan University, PRC. Among the 850 healthy controls, 306 were female and 544 were male, with an average age of 42.9 years. 440 periodontal patients (180 females and 260 males, aged 28 to 65 years) with moderate to severe chronic periodontitis were recruited from West China Hospital of Stomatology, Sichuan University, PRC. The data of the recruited subjects are presented in Table 1.

**Table 1:** The clinical data (Mean ± SD) of control subjects and periodontitis patients

Parameters	Control subjects (N = 850)	Periodontitis patients (N = 440)
Age (years)	42.9 ± 9.7	49.3 ± 13.6
Age range (years)	26 - 60	28 - 65
Male / female	544 / 306	260 / 180
PD (mm)	2.7 ± 1.2	6.1 ± 2.7*
Sites% with BOP	40.3 ± 9.5	78.2 ± 19.8*
Sites% with gingival recession	1 ± 1.2	38.9 ± 25.9*
Sites% with calculus	34.1 ± 13.6	63.0 ± 25.8
CAL (mm)	0.0	6.2 ± 2.9

PD: probing depth; BOP: bleeding on probing; CAL: clinical attachment loss.

Significant difference from the control subjects, *p < 0.05.

The sample size of this study was determined based on the reports of Machin and Lemeshow. In the report, the sample size was calculated based on 0.05 level of significance for two arms to achieve 90% power [47,48]. The study protocols were approved by the Ethics Committee, West China Hospital of Stomatology, Sichuan University. Informed consent was obtained from all subjects.

The diagnosis of CP was made following the criteria defined by the American Academy of Periodontology in 1999 [49]. The periodontal disease classification system forms the basis for the criteria of diagnosis of periodontitis [2,49,50].

All subjects were free of oral soft tissue abnormalities or severe dental caries. An intra-oral examination of periodontal conditions including supragingival/subgingival calculus, gingival recession, bleeding on probing (BOP), probing depth (PD), clinical attachment loss (CAL), gingival recession and tooth mobility was performed. The diagnostic criteria for periodontitis were referred to the 1999 International Classification of the Periodontal Disease and Conditions [49]. Subjects who were examined and determined to be free of periodontal disease, gingival recession, CAL and probing depth of greater than 3 mm were regarded as periodontally healthy. If subjects presented with probing depth of greater than 5 mm, CAL greater than 4 mm, some degree of gingival recession and tooth mobility, they were regarded to be severe chronic periodontitis patients [13,23,31].

### Preparation of control and patient blood samples

Peripheral blood samples were collected by direct venipuncture from the arm vein of each subject: 20 ml in lithium heparin tubes and 10 ml in clot blood tubes (BD Vacutainer, NJ USA). The samples were certrifuged for 10 min at 1,500 rpm, and serum and plasma was then collected for enzyme linked immunosorbent assay (ELISA) analysis. The remaining cellular components were transferred to a 50 ml centrifuge tube and an additional 45 ml red blood cell lysis buffer was added. The tube was inverted several times and then centrifuged for 10 min at 1,500 rpm. The supernatants were discarded, and the remaining components were washed with 0.9% PBS used for DNA extraction.

### Extraction of DNA from samples and polymerase chain reaction (PCR) to amplify the polymorphic site

Genomic DNA was extracted from blood samples following the blood protocols from the QIAamp DNA Blood Mini Kit (QIAGEN, MD, USA). The concentration of DNA was estimated by measurements of OD_260_ by a spectrophotometer (U1800, Hitachi, Japan). Extracted DNA was labeled and stored at -80°C until use.

A PCR kit (Promega Corporation, U.S.A), consisting of nuclease-free water and PCR Master Mix was used according to the manufacturer’s instructions. The PCR Master Mix includes 50 units/ml *Taq* DNA Polymerase supplied in a proprietary reaction buffer (pH 8.5), 400 μM of dATP, dGTP, dCTP, and dTTP, in addition to 3 mM of MgCl_2_. All procedures were carried out in a sterile and stable environment to prevent external contamination.

PCR was undertaken in a thermal cycler (MJ, U.S.A.) with a mixture containing 20 units of nuclease-free water, 25 units of PCR Master Mix, 0.5 units of each designed cytokine genes primer (Invitrogen, USA) (Table 2 &3). All primers were designed using the Roche UPL Primer Design Program, and 3 units of the extracted DNA sample were mixed to undergo thermal cycling. The thermal cycler was applied for PCR amplification of the DNA samples according to conditions set out in Table 2[13,23,31]. All products from thermal cycling were labeled accordingly and stored at -80°C until use. In this study, β-actin was used (Table 2) with the forward primer 5’-CCTCTATGCCAACACAGTGC-3’and reverse primer 5’-ATACTCCTGCTTGCTGATCC -3’. β-actin is considered to be a constitutive housekeeping gene for PCR, having been used to compare changes in specific gene expressions [51].

**Table 2:** PCR conditions for various genes

Gene	Product size (bp)	Denaturation	Annealing	Extension	Cycles
IL-1α	229	94°C,1min	55°C, 30s	72°C, 60s	35
IL-1β	305	94°C, 5mins	56°C, 45s	72°C, 60s	35
IL-6	296	95°C, 60s	60°C, 60s	72°C, 60s	35
TNF-α	133	94°C, 1min	61°C, 1min	72°C, 60s	35
IFN-γ	366	95°C, 5mins	56°C, 30s	72°C, 5mins	30
IL-4	195	95°C, 5mins	51°C, 60s	72°C, 60s	35
IL-10	139	94°C, 30s	60°C, 45s	72°C, 60s	35
β-actin	211	94°C, 30s	55°C, 30s	72°C, 60s	30

**Table 3:** The primer sequences and restriction enzymes used for detection of cytokine DNA polymorphism genes

Cytokines	Primers	Sequence	Position	Restriction Enzyme	Digestion Time (hours)	References
IL-1α	Forward	5'ATGGTTTTAGAAATCATCAAGCCTAGGCA-3'	-889	*Fnu*4H1	>12	Walker *et al.*
	Reverse	5'AATGAAAGGAGGGGAGGATGACAGAAATGA-3'				
IL-1β	Forward	5'-TGGCATTGATCTGGTTCATC-3'	-511	*Aval*	>12	Néstor *et al*.
	Reverse	5'-GTTTAGGAATCTTCCCACTT-3'				
IL-6	Forward	5'TTGTCAAGACATGCCAAGTGCT-3'	-174	*Nla* III	4	Trevilatto *et al*.
	Reverse	5'-GCCTCAGAGACATCTCCAGTCC-3'				
TNF-α	Forward	5'-GAAGCCCCTCCCAGTTCTAGT TC-3'	-238	*Ava*II	4	Sleijffers *et al.*
	Reverse	5'-CACTCCCCATCCTCCCTGGTC-3'				
IFN-γ	Forward	5'-GCTGTCATAATAATATTCAGAC-3'	-874	*Alw*	4	Inoue *et al*
	Reverse	5'-CGAGCTTTAAAAGATAGTTCC-3'				
IL-4	Forward	5'-TAAACTTGGGAGAACATGGT-3'	-590	*Ava* II	>12	Scarel-Caminaga *et al.*
	Reverse	5'-TGGGGAAAGATAGAGTAATA-3'				
IL-10	Forward	5'-CTCGCTGCAACCCAACTGGC-3'	-1082	*Mnl* I	4	Chin *et al.*
	Reverse	5'-TCTTACCTATCCCTACTTCC-3'				

### Restriction digest using *Fnu4H1*, *Aval*, *Nla III*, *Alw*, *Mnl I* and *AvaII* for IL-1α, IL-1β, IL-6, IFN-γ, IL-10, and TNF-α and IL-4 respectively

The amplified fragments generated from the PCR procedure were digested: 1) the 229 bp fragment on IL-1α was recognized by *Fnu4H1* (Fermentas Life Sciences, U.S.A.) [52], 2) the 305 bp fragment on IL-1β was recognized by *Aval* (Fermentas Life Sciences, U.S.A.) [53], 3) the 296 bp fragment on IL-6 was recognized by *NlaIII* (Fermentas Life Sciences, U.S.A.), [54] 4) the 296 bp fragment on IFN-γ was recognized by *Alw* (Fermentas Life Sciences, U.S.A.) [55], 5) the 139 bp fragment on IL-10 was recognized by *Mnl I* (Fermentas Life Sciences, U.S.A.) [56], and 6) both the 296 bp fragment on TNF-α and 195 bp fragment on IL-4 were recognized by *AvaII* (Fermentas Life Sciences, U.S.A.) [57,58].

For each digest, 10 μl of the amplified PCR product was mixed with 2.5 to 5 units of the corresponding restriction enzyme, 10 μl of nuclease-free water and 0.5 to 1 μl of restriction enzyme buffer. The entire mixture was incubated for more than 4 hours at 37°C (Table 3) [13,23,31]. All digestion reagents were kept on ice before incubation to prevent denaturation. Every sample was digested twice to ensure for consistency of the amplicions.

### Electrophoresis and visualization of digest product

The 10 μl of digestion product and 1 μl of Ready-Load 1 Kb DNA Ladder (Invitrogen, Spain) were loaded into 2-4% agarose gel (Invitrogen, Spain) containing 0.5 μg/ml of ethidium bromide [13,23,31]. The gel underwent electrophoresis at 100 volts and 100 milliAmperes for 30 minutes. Afterward, the gel was visualized using a Dolphin-DOC ultraviolet illuminator (Wealtec, South Africa).

### Sera measured by ELISA

ELISA was performed using the serum samples, following the manufacturer’s instructions from cytokines ELISA Kit (Diaclone, France) [59]. The normal detection ranges of biomarkers are detailed in Table 4. The procedures were as follows: 100 μl of the standard group solutions and serum of each subject were pipetted into a 96-well microplate. The plate was incubated for 2 to 3 hours at 350 rpm and washed with washing buffer three times. Then the wells were dried and 200 μl of substrate tetramethylbenizidine was added into each well for 20 min in the dark at room temperature. The plates were read at 450 nm wavelength using Universal Microplate Reader (Sunrise, TECAN, Austria). The levels of cytokines in the samples were obtained by comparison with the standard curve generated from standards supplied by the manufacturer [59].

**Table 4:** Complete blood count (Mean ± SD) of control subjects (N = 850) and periodontitis patients (N = 440)

Parameters	Control subjects	Periodontitis patients	Normal Range	Unit
White blood cell	6.33 ( ± 1.31)	3.96 (±1.02)	4.00 - 11.00	10^9^/L
Red blood cell	4.52 ( ± 0.13)	4.25 (±0.10)	3.8 - 6.0	10^12^/L
Hemoglobin	15.0 ( ± 0.50)	12.5 (±0.43)	11.5 - 16.5	g/dL
Platelet	273 ( ± 27.12)	241 (±23.31)	150 - 400	10^9^/L
Neutrophils	4.45 ( ± 1.05)	*1.68 (±0.71)	2.0 - 7.5	10^9^/L
Lymphocytes	1.37 ( ± 0.64)	*1.42 (±0.26)	1.30 - 3.5	10^9^/L
Monocyte	0.34 ( ± 0.19)	0.18 (±0.11)	0.2 - 0.7	10^9^/L
Eosinophil	0.12 ( ± 0.60)	0.06 (±0.02)	0.0 - 0.5	10^9^/L
Basophil	0.05 ( ± 0.03)	0.02 (±0.01)	0.0 - 0.1	10^9^/L
Neutrophils	70.3 ( ± 13.42)	*49.9 (±11.43)	4.0 - 75.0	%
Lymphocytes	21.6 ( ± 9.06)	*42.3 (±10.69)	20 - 45	%
Monocyte	5.4 ( ± 3.02)	5.4 (±2.89)	4.7 - 12.2	%
Eosinophil	1.9 ( ± 0.34)	1.8 (± 0.52)	0.7 - 7.0	%
Basophil	0.8 ( ± 0.12)	0.6 (± 0.24)	0.1 - 1.2	%

Significant difference from the control, * *p* < 0.05

### Statistical analysis

The Chi-squared test was applied to examine the differences in genotype distribution, allele frequency and carriage rate between healthy and patient groups. The alleles were calculated as an odds ratio (OR) with 95% confidence interval (95% CI) [13,23,31]. The soluble protein levels of cytokines were evaluated with an independent t-test [59], and p < 0.05 was considered statistically significant. Statistical analysis was performed using SPSS 18.0 for windows (SPSS Inc., U.S.A.).

## Results

The clinical parameters of the healthy subjects and periodontitis patients were shown in Table 1. The periodontitis patient group exhibited a significantly greater mean of PD (6.1 ± 2.7 mm vs. 2.7 ± 1.2 mm), CAL (6.2 ± 2.9 mm vs 0.0) and a higher percentage of sites with BOP (78.2 ± 19.8% vs. 40.3 ± 9.5%) and gingival recession (38.9 ± 25.9% vs. 1 ± 1.2%) than the control group (*p* < 0.05). There was no significant difference found in the age and gender ratio between the two groups.

The total blood count of the healthy control subjects and periodontitis patients are presented in Table 4. With regard to the clinical parameters, the results of all subjects were within the normal range, although the patient group exhibited a relatively higher count and percentage of lymphocytes, and a relatively lower count and percentage of neutrophils (*p* < 0.05) compared to the periodontally healthy controls.

The protein expression of patient and control samples for IL-1α, IL-1β, IL-6, TNF-α, IFN-γ, IL-10, and IL-4 (Table 5) measured by ELISA confirmed a statistically significant difference (p < 0.001). The digestion of fragments of various genes examined under a gel documentation system (Wealtec, South Africa) showed that the pro-inflammatory cytokines IL-1α and TNF-α, and the anti-inflammatory cytokines IL-4 and IL-10 demonstrated an association with chronic inflammation in patients (X^2^: p < 0.0001).

**Table 5:** Protein expression (Mean ± SD) of various cytokines in patient and control groups as measured by ELISA

Cytokines	Control subjects (N = 850)	Periodontitis patients (N = 440)	*p* value	Detection Range pg/ml
IL-1α	24.08 ( ± 4.62)	59.73 ( ± 11.36)	0.0008	31.25 - 1000
IL-1β	19.57 ( ± 3.75)	78.07 ( ± 17.09)	0.0004	15.6 - 500
IL-6	27.35 ( ± 2.87)	92.94 ( ± 17.44)	0.0005	2 - 200
TNF-α	26.90 ( ± 4.55)	67.61 ( ± 14.60)	0.0002	8 - 800
IFN-γ	52.77 ( ± 8.12)	94.71 ( ± 9.90)	0.0003	5 - 400
IL-4	1.16 ( ± 0.20)	4.62 ( ± 0.58)	0.0003	0.7 - 35
IL-10	22.70 ( ± 3.41)	46.89 ( ± 5.85)	0.0012	5 - 400

After digestion by *Fnu4H1*, IL-1α formed the DNA products of 153 and 76 bp for homozygous C allele, and 124, 76 and 29 bp for homozygous T alleles [52]. The detection frequency of the homozygous C allele of IL-1α was similar in the subjects with chronic periodontitis (62%) and control group (55%) (X^2^, p = 0.18), but in the C/C genotype, the periodontitis patients presented a frequency of 52%, compared to 20% in the control group. The odds ratio for carriage of IL-1α allele (T/T + C/T genotypes combined to compare with the C/C genotype) was 4.368 (95% CI = 2.309 - 8.264, X^2^: p < 0.0001) in periodontitis patients (Table 6).

**Table 6:** Genotype and allele frequency of cytokines in periodontitis patients (CP) and control subjects

Genotypes	CP Patients *n* = 440 (%)	Healthy subjects *n* = 850 (%)	CP versus Controls		Alleles	CP patients *n* = 880 (%)	Healthy subjects *n* = 1700 (%)	CP versus Controls	
							
			OR (95% CI)	*X^2^* *p* values				OR (95% CI)	*X^2^* *p* values
**IL-1α**									
*C/C	**229(52)**	**170(20)**	**4.3681**	**23.32**	**C**	**546(62)**	**935(55)**	**1.13597**	**1.78**
T/T	123(28)	85(10)	**(2.3089-8.2638)**	**<0.0001**	T	334(38)	765(45)	**(0.865-2.1374)**	**0.1822**
C/T	88(20)	595 (70)							
**IL-1β**									
*T/T	**128(29)**	**314(37)**	**0.7132**	**0.97**	**T**	**510(58)**	**986(58)**	**0.9974**	**0**
C/C	61(14)	179(21)	**(0.3624-1.4035)**	**0.3247**	C	370(42)	714(42)	**(0.6393-1.5559)**	**1.000**
C/T	251(57)	357(42)							
**IL-6**									
*G/G	**110(25)**	**408(48)**	**0.3595**	**8.57**	**G**	**510(58)**	**1037(61)**	**0.8781**	**0.33**
C/C/	25(8)	221(26)	**0.177-0.7301**	**0.0034**	C	370(42)	663(39)	**(0.5626-1.3706)**	**0.5657**
G/C	295(67)	221(26)							
**TNF-α**									
*G/G	**189(43)**	**145(17)**	**3.7156**	**17.47**	**G**	**502(57)**	**578(34)**	**2.5054**	**17.23**
A/A	132(30)	408(48)	**(1.9432-7.1043)**	**0.0001**	A	378(43)	1122(66)	**(1.6056-3.9095)**	**0.0001**
A/G	119(27)	297(35)							
**IFN-γ**									
*T/T	**251(57)**	**476(56)**	**1.0382**	**0.010.9203**	**T**	**580(66)**	**1105(65)**	**1.0475**	**0.04**
A/A	106(24)	221(26)	**(0.5548-1.9427)**		A	300(34)	595(35)	**(0.6594-1.664)**	**0.8415**
T/A	83(19)	153(18)							
**IL-4**									
*C/C	**9(2)**	**230(27)**	**16.0032**	**13.2**	C	211(24)	935(55)	3.8369	30.16
T/T	242(55)	153(18)	**(2.1784-117.5633)**	**0.0003**	T	669(76)	765(45)	(2.3075-6.3797)	0.0001
T/C	189(43)	467(55)							
**IL-10**									
*A/A	**277(63)**	**782(92)**	**6.5546**	**5.211**	**A**	**590(67)**	**1581(93)**	**6.5731**	**63.26**
G/G	132(30)	51(6)	**(3.2316-13.2948)**	**0.0001**	G	290(33)	119(7)	**(3.9186-11.0259)**	0.0001
A/G	31(7)	17(2)							

IL-1α: T/T+C/T versus C/C; IL-1β: C/C+ C/T versus T/T;

IL-6: C/C+G/C versus G/G; TNF-α: A/A+A/G versus G/G;

IFN-γ: A/A+T/A versus T/T; IL-4: T/T +T/C versus C/C;

IL-10: G/G+A/G versus A/A

OR, odds ratio; CI, confidence interval;

Significant difference from the control, *p < 0.05

After digestion by *AvaII*, TNF-α formed the DNA products of 70 and 63 bp for homozygous G alleles, and 63, 49 and 21 bp for homozygous A alleles.^81^ The detection frequency of the homozygous G allele of TNF-α was higher in the subjects with chronic periodontitis (57%) than the control group (34%) (X^2^: p < 0.0001, OR: 2.505, CI = 1.606 - 3.910). Comparing the G/G genotype, periodontitis patients presented higher a frequency of 43% than 17% in the healthy controls (p < 0.0001). The odds ratio for carriage of TNF-α allele (A/A and A/G genotypes combined to compare with the G/G genotype) was 3.716 (95% CI = 1.943 - 7.104) in periodontitis patients (Table 6).

The homozygous T alleles of IL-4 were represented by a DNA band with a size of 195 bp, homozygous C alleles were represented by DNA bands with sizes of 18 and 177 bp [58]. The detection frequency of the homozygous T alleles of IL-4 was higher in the patient (76%) than control group (45%). The Chi-Square test result was *p* < 0.0001, and the odds ration was 3.837 (95% CI = 2.308 - 6.380) between the patient and control group. Comparing T/T genotype, the periodontitis patients presented a higher frequency of 55% in contrast to 18% in periodontally healthy controls (p < 0.001). The odds ratio for carriage of IL-4 allele (T/T and C/T genotypes combined compared with the C/C genotype) was 16.003 (95% CI = 2.178 - 117.563) in periodontitis patients (Table 6).

The homozygous A alleles of IL-10 were represented by a DNA band with a size of 139 bp, and homozygous G alleles were represented by DNA bands with sizes of 106 and 33 bp. The detection frequency of the homozygous A alleles of IL-10 was higher in periodontally healthy controls (93%) than the patient group (67%). The result of the Chi-square test was p < 0.001, and the odds ratio was 6.573 (95% CI = **3.919** - 11.026) between the disease and control group. Comparing A/A genotype, the periodontitis patients presented lower frequency of 63% than 92% in control (p < 0.001). The odds ratio for carriage of IL-10 allele (G/G and A/G genotypes combined compared with the A/A genotype) was 6.555 (95% CI: 3.232-13.265) in periodontitis patients (Table 6).

Other genes investigated in the patients and healthy subjects (IL-1β, IL-6, IFN-γ and IL-10) did not show any significant difference (p > 0.05), and the lower bound of confidence interval was below 1.

Overall, significant difference was found in the distribution of IL-1α, TNF-α, IL-4 and IL-10 between the two groups (Table 6). For IL-1α, the detection frequency of C allele was 62% in the patients group and 55% in the control. The subjects with the C allele are at similar risk for both groups. The genotype of C/C was significantly higher in the patient group (52%) than in the controls (20%) (*p* < 0.001). Individuals with the C/C genotype are at over four times higher risk for moderate to severe periodontitis than people with T/T and C/T genotypes (*p* < 0.001, OR = 4.368 with 95% CI 2.309 - 8.264). The detection frequency of G allele in TNF-α was 57% in the patients group and 34% in the control group. The patients with the G allele are at two-fold risk for the disease. The genotype of G/G was significantly higher in the patient group (43%) than in the controls (17%) (*p* < 0.001). Individuals with the G/G genotype are at nearly four times higher risk for moderate to severe periodontitis than persons with A/A and A/G genotypes (*p* < 0.001, OR = 3.716 with 95% CI 1.943 - 7.104). In IL-4, the detection frequency of C allele was 24% in the patient group and 55% in the healthy control group. The control subjects with the C allele are at almost four times less risk for the disease. The genotype of C/C was significantly lower in the patients (2%) compared to the healthy controls (27%) (*p* < 0.001). Individuals with the C/C genotype are at over sixteen times higher risk for moderate to severe periodontitis than persons with T/T and C/T genotypes (*p* < 0.001, OR = 16.003 with 95% CI = 2.178 - 117.563). The detection frequency of A allele in IL-10 was 67% in the patient group and 93% in the healthy control group. The subjects with the A allele are at six-fold less risk to the disease. The genotype of A/A was significantly lower in the patients group (63%) than in the controls (92%) (*p* < 0.001). Individuals with the A/A genotype are over six times at higher risk for moderate to severe periodontitis than persons with G/G and A/G genotypes (*p* < 0.001, OR = 6.555 with 95% CI = 3.232 - 13.295).

## Discussion

The levels of the proinflammatory cytokines IL-1α, IL-1β, TNF-α, IFN-γ and IL-6 are characteristically increased in diseased periodontal tissues or gingival crevicular fluid [60-63]. On the contrary, the initiation and progression of periodontal inflammation may be due to a lack or inappropriate response of the anti-inflammatory cytokines IL-4 and IL-10 in chronic periodontitis [61-63]. The role of polymorphisms in host responses in the progression of periodontitis has been well documented in scientific literature. In most situations, the genetic polymorphisms cause a change in the protein or its expression altering the immune response.

The results presented here demonstrate a similar frequency of C and T alleles of IL-1α -889 in CP and control groups. The frequency of the C allele in CP patients was 62% which is comparable to other studies in some populations [64]. The latter demonstrates an important issue that genetic polymorphism vary among different ethnic populations, because the carriage rate of C allele tend to be lower in other Asian populations than the Chinese subjects in our study [65,66]. The frequency of T allele, in contrast, was higher in the control than CP group. The IL-1α CC genotype (OR = 4.368; 95% CI = 2.309 to 8.264) was significantly associated with CP. This is in accordance with other similar studies [67]. In this study, IL-1β -511 failed to demonstrate an association with periodontitis, which corresponds to four other studies to date [68,69]. The carriage rate of rare allele was the same in the patient and control groups. In all other previous reports, this rate was similar.

Another proinflammatory cytokine, TNF-α, possesses a wide range of immunoregulatory functions, including production of secondary mediators [70]. Many studies have investigated the SNPs in the promoter region at positions -1031, -863, -857, -376, -308, and -238. Among the above polymorphic sites, position -308 has been most researched, although most studies failed to demonstrate an association with periodontitis. However, the present study managed to showed an association of TNF-α GG genotype (OR = 3.716; 95% CI = 1.943 - 7.104) with periodontitis. Our studied cases showed a higher frequency of homozygous (G/G) genotype compared to healthy controls, which could be considered a risk genotype for periodontitis susceptibility.

In the current study, IL-6 did not exhibit an association with periodontitis, in contrast to studies on Caucasians and Brazilians [31,37]. The carriage rate of both C and G alleles was similar in CP and control groups. However, the total cases showed a significantly higher frequency of heterozygous genotype IL-6 -174 (G/C) compared to the periodontally healthy controls. Thus, IL-6 -174 G/C genotype may be considered a risk genotype for periodontitis susceptibility.

The IL-4 -590 promoter polymorphism is the most studied polymorphisms of IL-4. Despite so, case-control studies have not shown any relationship between the IL-4 gene polymorphism and susceptibility to chronic periodontitis [71,72]. In contrast, our results demonstrated an association of IL-4 C/C genotype (OR = 16.003; 95% CI = 2.178 - 117.563) with a lower frequency in periodontitis patients. The T/T genotype, however, was more prevalent in our patients with periodontitis (55%), suggesting that this may be a risk genotype for periodontitis susceptibility. A reduction in the frequency of the C/C genotype in patients confers a protective influence against the development of the disease.

The IL-10 -1082 polymorphism was evident in our results, displaying a lower frequency of the A/A genotype in periodontitis patients than the healthy controls (63% versus 92%) [32-34]. A higher frequency of IL-10 -1082 G/G genotype was found in periodontitis patients (30%), compared to the periodontally healthy controls.(6%). The results of the clinical trial were in accordance with previous reports: the proportion of subjects exhibiting the G/G genotype was significantly higher in subjects with severe periodontitis than in periodontally healthy individuals [73]. The incongruous results can be explained by the racial difference or association with other interactive types of cytokines or genetic markers predisposing for the disease in the studied cases.

## Conclusions

The cytokine gene polymorphisms may be used as a marker for periodontitis susceptibility, clinical behaviour and severity. This detection offers early diagnosis and induction of prophylaxis to other family members against disease progression.

## Competing interests

We declare that we have no financial and personal relationships with other people or organizations that can inappropriately influence our work, there is no professional or other personal interest of any nature or kind in any product, service and/or company that could be construed as influencing the position presented in, the article entitled, “Gene polymorphism and protein of human pro- and anti-inflammatory cytokines in Chinese healthy subjects and chronic periodontitis patients”.

## Authors' contributions

WTYL conducted the research, performed data collection and data analysis, and participated in manuscript writing. YY and CF, JL and YT conducted the clinical examination and performed data collection. LB performed data collection and data analysis. MW supervised clinical examination and participated in manuscript planning. HL performed data analysis and participated manuscript writing. MNBC conducted the clinical examination and participated in manuscript writing. LWCC participated in manuscript planning and writing. Dr. Liu Qing was responsible for data collection. All authors read and approved the final manuscript.
